# In vitro and in vivo differences in neurovirulence between D614G, Delta And Omicron BA.1 SARS-CoV-2 variants

**DOI:** 10.1186/s40478-022-01426-4

**Published:** 2022-09-05

**Authors:** Lisa Bauer, Melanie Rissmann, Feline F. W. Benavides, Lonneke Leijten, Peter van Run, Lineke Begeman, Edwin J. B. Veldhuis Kroeze, Bas Lendemeijer, Hilde Smeenk, Femke M. S. de Vrij, Steven A. Kushner, Marion P. G. Koopmans, Barry Rockx, Debby van Riel

**Affiliations:** 1 Department of Viroscience, Erasmus Medical Center, Rotterdam, The Netherlands; 2 Department of Psychiatry, Erasmus Medical Center, Rotterdam, The Netherlands

**Keywords:** Neurovirulence, Neuroinvasion, Neurotropism, Coronavirus, SARS-CoV-2, COVID-19, Microglia activation, Brain olfactory bulb, Interferon

## Abstract

Severe acute respiratory syndrome coronavirus 2 (SARS-CoV-2) infection is associated with various neurological complications. Although the mechanism is not fully understood, several studies have shown that neuroinflammation occurs in the acute and post-acute phase. As these studies have predominantly been performed with isolates from 2020, it is unknown if there are differences among SARS-CoV-2 variants in their ability to cause neuroinflammation. Here, we compared the neuroinvasiveness, neurotropism and neurovirulence of the SARS-CoV-2 ancestral strain D614G, the Delta (B.1.617.2) and Omicron BA.1 (B.1.1.529) variants using in vitro and in vivo models. The Omicron BA.1 variant showed reduced neurotropism and neurovirulence compared to Delta and D614G in human induced pluripotent stem cell (hiPSC)-derived cortical neurons co-cultured with astrocytes. Similar differences were obtained in Syrian hamsters inoculated with D614G, Delta and the Omicron BA.1 variant 5 days post infection. Replication in the olfactory mucosa was observed in all hamsters, but most prominently in D614G inoculated hamsters. Furthermore, neuroinvasion into the CNS via the olfactory nerve was observed in D614G, but not Delta or Omicron BA.1 inoculated hamsters. Furthermore, neuroinvasion was associated with neuroinflammation in the olfactory bulb of hamsters inoculated with D614G. Altogether, our findings suggest differences in the neuroinvasive, neurotropic and neurovirulent potential between SARS-CoV-2 variants using in vitro hiPSC-derived neural cultures and in vivo in hamsters during the acute phase of the infection.

**Supplementary Information:**

The online version contains supplementary material available at 10.1186/s40478-022-01426-4.

## Introduction

The severe acute respiratory syndrome coronavirus 2 (SARS-CoV-2) causing coronavirus disease 2019 (COVID-19) is associated with a wide range of neurological complications in the acute and post-acute stages. In the acute stage, symptoms include loss of smell (anosmia), headache, fatigue, seizures, confusion and cerebrovascular deficits [1, 2]. Although the incidence estimates vary across studies, a substantial proportion of patients suffer from neurological manifestations in the acute phase of disease [3, 4]. Neuropsychiatric complications such as depression, anxiety and cognitive problems may also persist in the post-acute stage, which are often observed in patients suffering from Long Covid [5–8]. The underlying mechanisms of the neurological symptoms remain poorly understood, but given the diversity of complications, multiple mechanisms are likely to contribute.

Currently, it is unclear whether (emerging) SARS-CoV-2 variants differ in their neuroinvasiveness, neurotropism and neurovirulence. However, several studies have shown that in mice and hamsters respiratory disease is less severe after inoculation with Omicron BA.1 variant compared to earlier variants [9–12], and epidemiological data suggest that hospitalization rates are lower with the Omicron BA.1 variant compared to earlier SARS-CoV-2 variants [13, 14]. Indirect evidence suggest that neurological complications in humans might be less common after infection with Omicron BA.1 compared to earlier variants such as D614G or the Delta variant, since both anosmia and Long Covid occur less frequently, although this might be—in part—related to pre-existing immunity [15–18]. A recent study in transgenic mice—in which ACE-2 expression is higher in the brain compared to the human brain [19, 20] —shows frequent neuroinvasion and neurovirulence of D614G and Delta variant but not Omicron BA.1 [21]. In summary, these studies suggest that there might be differences in the neuroinvasiveness, neurotropism and neurovirulence among SARS-CoV-2 variants.

Several studies have shown that SARS-CoV-2 might enter the central nervous system (CNS) via different routes, of which the olfactory nerve appears an important one [22, 23]. The olfactory nerve connects the olfactory mucosa directly with the olfactory bulb in the brain providing an efficient anatomical entry point from the nasal cavity into the brain [24]. Once within the CNS, the neurotropism of SARS-CoV-2 appears to be restricted to a subset of permissive CNS cells based on in vivo (human, hamster, non-human primates, ferrets) [22, 23, 25–30] and in vitro [30–38] studies. So far, efficient replication of SARS-CoV-2 has only been observed in choroid plexus epithelial cells in vitro [31, 33]. SARS-CoV-2 inoculation in transgenic hACE-2 mice resulted in efficient neuroinvasion, and subsequent spread throughout the CNS [21], which was not observed in mice that received adenovirus-associated vectors that induce ACE-2 expression in the respiratory system only [39]. This suggest that the efficient neuroinvasion and spread throughout the CNS observed in the hACE-2 transgenic mice was related to the higher expression of ACE-2 in the CNS of these mice compared to human CNS [19, 20].

In order to understand if SARS-CoV-2 variants differ in their neuroinvasiveness, neurotropism and neurovirulence, we investigated these characteristics of the ancestral D614G variant, as well as Delta and Omicron BA.1 variant, in human induced pluripotent stem cell (hiPSC) derived cortical neurons in vitro and Syrian golden hamsters in vivo. Furthermore, we quantified the neuroinvasive, neurotropic and neurovirulent potential of the ancestral D614G variant, Delta and Omicron BA.1 variants in the olfactory bulb, cerebral cortex and cerebellum of experimentally inoculated Syrian golden hamsters.

## Material and methods

### Cells

VeroE6 (ATCC CRL 1586) cells were maintained in Dulbecco’s modified Eagle’s medium (DMEM; Lonza,) supplemented with 10% fetal calf serum (FCS; Sigma-Aldrich,), 10 mM HEPES, 1.5 mg/ml sodium bicarbonate, 100 IU/ml penicillin (Lonza,), and 100 μg/ml streptomycin (Lonza). Calu-3 cells were cultured in Opti-MEM I (1) + GlutaMAX (Gibco) supplemented with 10% FBS, penicillin (100 IU/mL), and streptomycin (100 IU/mL). Both cells were grown at 37 °C in a humidified CO_2_ incubator and routinely tested for mycoplasma.

### hiPSC cell line

Human induced pluripotent stem cells (iPSCs), were used to generate glutamatergic neurons (WTC-11 Ngn2). hiPSC were maintained in hiPSC medium (Table 1) and passaged when confluency of 80% was reached. For passaging, hiPSC were washed with PBS and released with Accutase (Life Technologies) and plated on Matrigel-coated 6-wells. For coating, Matrigel (Corning, 10 μl/ml) was resuspended in Knock-Out Dulbecco’s Modified Eagle Medium (KO DMEM; ThermoFisher Scientific) and plates were incubated for 60 min at 37 °C. Medium was refreshed every other day, and cells were cultured at 37 °C and 5% CO_2_.

**Table 1:** List of differentiation media

Name	Reagents with final concentration	Manufacturer
hiPSC medium	Stemflex medium	ThermoFisher Scientific
	100 IU/ml penicillin	Lonza
	100 µg/ml streptomycin	Lonza
	10 µl/ml fresh RevitaCell	ThermoFisher Scientific
Differentiation medium	Advanced DMEM/F12 medium	ThermoFisher Scientific
	100 IU/ml penicillin	Lonza
	100 µg/ml streptomycin	Lonza
	0.1 mM non-essential amino acids	Lonza
	1% N2 supplement	ThermoFisher Scientific
	10 ng/ml fresh Human Recombinant Neurotrophin-3 (NT3)	Stemcell Technologies
	10 ng/ml fresh brain-derived neurotrophic factor (BDNF)	Prospecbio
	200 ng/ml fresh laminin	Corning
	4 µg/ml fresh Doxycycline	Sigma
Ngn2 medium	Neurobasal medium	ThermoFisher Scientific
	100 IU/ml penicillin	Lonza
	100 µg/ml streptomycin	Lonza
	2 mM glutamine	Lonza
	2% B27 minus RA supplement	ThermoFisher Scientific
	10 ng/ml fresh Human Recombinant Neurotrophin-3 (NT3)	Stemcell Technologies
	10 ng/ml fresh brain-derived neurotrophic factor (BDNF)	Prospecbio
	4 µg/ml fresh Doxycycline (DOX)	Sigma

### hiPSC-derived Ngn2 neurons and astrocytes

iPSCs were directly differentiated into excitatory cortical layer 2/3 neurons by doxycycline induced overexpression of neurogenin-2 (Ngn2) as described previously [40, 41]. In short, coverslips were coated with poly-l-ornithine (Sigma, 100 μg/ml) for 1 h at room temperature in the dark. Afterwards coverslips were washed three times with sterile water and air- dried for 30 min. A droplet of Matrigel (Corning, 10 μl in 1 ml KO DMEM, Life Technologies) was placed in the middle of the coverslip and incubated for 1 h at 37 °C. At day 0, hiPSCs were placed on coated coverslip and incubated for 30 min at 37 °C with 5% CO_2_ to allow cell attachment. After attachment wells were filled with hiPSC medium supplemented with doxycycline (4 µg/ml) (Table 1). On next day (Day 1), the medium was refreshed with differentiation medium (Table 1). In order to guarantee the formation of functional synapses and thus functional synaptic plasticity within the network, hiPSC-derived astrocytes were added to the culture in a 1:1 ratio. Human astrocytes were differentiated from neural progenitor cells that were made according to an embryoid-based protocol [42], from the WTC-11 human iPSC line (Coriell GM25256) [43]. The medium was refreshed the day after with Ngn2 medium (Table 1). During the differentiation and maturation, half of the medium was refreshed every other day. After 21 days the neural co-cultures were mature and used in experiments.

### Viruses

The SARS-CoV-2 D614G isolate (isolate BetaCoV/Munich/BavPat1/2020; European Virus Archive Global no. 026V-03883; kindly provided by C. Drosten) was propagated to passage three on Vero E6 cells in Opti-MEM I (1X) + GlutaMAX (Gibco), supplemented with penicillin (10,000 IU/mL) and streptomycin (10,000 IU/mL). The SARS-CoV-2 variants Delta (B.1.617.2) and Omicron BA.1 (B.1.1.529) were propagated to passage three on Calu-3 cells in Advanced DMEM/F12 (Gibco), supplemented with HEPES, Glutamax, penicillin (100 IU/mL) and streptomycin (100 IU/mL). The Delta and Omicron BA.1 sequences are available on GenBank under accession numbers OM287123 and OM287553, respectively. Sequencing of the virus stock did not reveal any major substitution as previously described [44]. Especially cell-culture adaptation in the polybasic cleavage side of the Spike protein were excluded. All three viruses were grown at 37 °C in a humidified CO_2_ incubator. Infections were performed at a multiplicity of infection (MOI) of 0.01 and virus was harvested after 72 h or at the peak of replication. The culture supernatant was stored at − 80 °C. Virus titers were determined by plaque assay as described below. All work was performed in a Class II Biosafety Cabinet under BSL-3 conditions at Erasmus Medical Center.

### Virus titrations

Ten-fold serial diluted samples were added to monolayers of Calu-3 cells and incubated for 4 h at 37 °C, washed once with PBS and then overlayed with 1.2% Avicel (FMC biopolymers) in Opti-MEM I (1X) + GlutaMAX for two days. Cells were fixed in 4% formalin for 20 min, permeabilized in 70% ice-cold ethanol and washed in PBS. Cells were incubated in 3% BSA (bovine serum albumin; Sigma) in PBS and stained with a rabbit anti-SARS-CoV-2-nucleocapsid antibody (Sino biological; 1:2000) in PBS containing 0.1% BSA, washed thrice in PBS, and stained with donkey anti-rabbit Alexa Fluor 488 (Invitrogen; 1:4000) in PBS containing 0.1% BSA. Cells were washed thrice in PBS and plates were scanned on the Amersham Typhoon Biomolecular Imager (channel Cy2; resolution 25 µm; GE Healthcare). All staining steps were performed at room temperature for one hour. Plaque assay analysis was performed using ImageQuant TL 8.2 software (GE Healthcare).

### Animals

#### Ethical statement

Research involving animals was conducted in compliance with the Dutch legislation for the protection of animals used for scientific purposes (2014, implementing EU Directive 2010/63) and other relevant regulations. The licensed establishment where this research was conducted (Erasmus MC) has an approved OLAW Assurance # A5051-01. Research was conducted under a project license from the Dutch competent authority and the study protocol (#17-4312) was approved by the institutional Animal Welfare Body. Animals were housed in groups of 2 animals in filter top cages (T3, Techniplast), in Class III isolators allowing social interactions, under controlled conditions of humidity, temperature and light (12-h light/12-h dark cycles). Food and water were available ad libitum. Animals were cared for and monitored (pre- and post-infection) daily by qualified personnel. All animals were allowed to acclimatize to husbandry for at least 7 days. For unbiased experiments, all animals were randomly assigned to experimental groups. The animals were anesthetized (3–5% isoflurane) for all invasive procedures. Hamsters were euthanized by cardiac puncture under isoflurane anesthesia and cervical dislocation.

#### Animals and experimental setup

Female Syrian golden hamsters (*Mesocricetus auratus*; 6 weeks old; Janvier, France) were handled in an ABSL-3 biocontainment laboratory. Groups of animals (n = 4) were inoculated intranasally with 1 × 10^5^ TCID_50_ D614G, 5 × 10^4^ PFU of Omicron BA.1 or Delta variants of SARS-CoV-2 or PBS (mock; n = 4) in a total volume of 100 µl per animal. Inoculation doses were adapted according to individual stock titres of each SARS-CoV-2 variant. On 5 dpi, infected animals were euthanized and the respiratory tract (nasal turbinate), as well as the CNS (olfactory bulb, cerebral cortex, cerebellum) was sampled for quantification of viral and genomic load, as well as for histopathology. The time point of 5 dpi was chosen to represent the peak of respiratory and clinical manifestation of disease. Furthermore, based on literature [28, 45] and our previous studies by Rissmann et al. [9] we assumed that the 5 dpi would facilitate both viral detection and evidence of inflammatory changes within the CNS. Mock infected animals were euthanized 14 days post mock infection. Samples for histopathological analyses were fixed in 10% formalin for 2 weeks, after which tissues were paraffin-embedded.

### Immunohistochemistry

For the detection of SARS-CoV-2, activated microglia (Iba-1) and CD3 antigen in tissues of olfactory bulb and nose of hamsters, 3 μm formalin-fixed, paraffin-embedded sections were deparaffinized, rehydrated and pre-treated by boiling for 15 min in citric acid buffer pH 6.0 (SARS-CoV-2) or TRIS–EDTA pH 9.0 (Iba-1 and CD3). Endogenous peroxidase was blocked with 3% hydrogen peroxide for 10 min at RT, after which slides were briefly washed with phosphate-buffered saline/0.05% Tween 20. Sections for SARS-CoV-2 IHC were blocked with 10% goat serum (X0907, DAKO, Agilent Technologies Netherlands B.V) for 30 min at RT. Slides were then incubated with a rabbit polyclonal antibody against SARS-CoV/SARS-CoV-2-nucleoprotein (40,143-T62, Sino Biological, Pennsylvania, USA) (1:1000), mouse CD3 (ab16669, Abcam, Cambridge, UK) (1:10, 20 µg/ml), human Iba-1 (019-19741, Wako Pure Chemical Corporation, Osaka, Japan) (1:200; 2.5 µg/ml)) or Rabbit IgG isotypecontrol (AB-105-C, R&D, UK) (1:200; 5 µg/ml) in PBS/0,1% BSA for 1 h at room temperature (RT). After washing, sections were incubated with peroxidase labeled goat-anti-Rabbit IgG (1:100) (P0448, DAKO, Agilent Technologies Netherlands B.V) in PBS/0,1% BSA for 1 h at RT. Peroxidase activity was revealed by incubating slides in 3-amino-9-ethylcarbazole (AEC) (Sigma) for 10 min, resulting in bright red precipitate, followed by counterstaining with hematoxylin. A lung section from an experimentally SARS-CoV-2 inoculated hamster and a brain and spleen section from a mock inoculated hamster were used as positive control for SARS-CoV-2, Iba-1 and CD3 staining respectively.

### SARS-CoV-2 in situ hybridization

BaseScope™ RNA probes were designed by Bio-Techne Ltd (Abingdon, UK) for BA-V-CoV-Wuhan-Nucleocapsid-3zz-st (846661). In situ hybridization was performed on formalin-fixed, paraffin-embedded consecutive sections from hamster olfactory bulb using BaseScope™ Reagent Kit v2–RED (323900) as described by the manufacturer.

### Counting of microglia

From every animal, three pictures of the granule cellular layer and the glomerular layer were taken. Topographical and histological specification of the anatomical location of the granule cell layer and the glomerular layer is shown in Additional File 1: Fig. S4. The number of IBA-1^+^ cells per high power field was determined by manual counting by two independent blinded observers. Averages and standard deviation of the counting was plotted.

### Microscopy

A 10 × air objective (Olympus) was used to select a region of interest with an Olympus BX51 microscope. Images were taken with a 200 × magnification (20 × air objective; Olympus) with CellSens software.

### Immunofluorescence staining

hiPSC-derived cortical neurons were fixed using 10% formalin for 30 min and afterwards washed with PBS. Cells were permeabilized for 15 min using 1% Triton X-100 in PBS which was followed by a blocking step with 0.5% Triton X-100 and 1% donkey serum (Sigma) in PBS for 30 min at room temperature. Primary antibody incubation was performed for 1 h at RT in PBS supplemented with 0.5% Triton X-100 and 1% bovine serum albumin (BSA) (antibody concentrations see Table 2). After washing with PBS, secondary antibody incubation was performed for 1 h at RT in PBS supplemented with 0.5% Triton X-100 and 1% bovine serum albumin (antibody concentrations see Table 2). To visualize cell nuclei, cells were incubated with a solution of Hoechst (Invitrogen, H3570) in PBS for 10 min before mounting. Slides were washed in PBS, dipped in water and mounted in ProLong Antifade Mountant (Thermo Fisher). Samples were imaged using a Zeiss LSM 700 confocal microscope.

**Table 2:** List of antibodies for immunofluorescence and flow cytometry

Antigen	Species	Dilution	Manufacturer	Cat #	Lot #
SARS-CoV-2 NP	Rabbit pAB	1:1000	Sino Biological	40143-T62	HD14JN0202
Iba-1	Mouse mAB	1:200	Wako Pure Chemical Corporation	019-19741	LKR1186
MAP-2	Guinea pig pAB	1:200	Synaptic Systems	188004	30021200
CD3	Rabbit mAB	1:10	Abcam	ab16669	GR3307114-8
Rabbit IgG	Rabbit pAB	1:200	R&D Systems	AB-105-C	ER1619081
Goat anti rabbit Ig-HRP	Goat pAB	1:100	DAKO	P044801	20079938

### Flow cytometry

Neural co-cultures, consisting of mature hiPSC-derived cortical neurons and hiPSC-derived astrocytes were washed with PBS, released with trypsin and collected in a V-bottom plate. The cells were fixed for 30 min in 10% formalin at room temperature. Afterwards, the cells were permeabilized using cytofix/cytoperm (BD Biosciences) for 20 min at 4 °C and blocked with 10% normal donkey serum for 30 min at 4 °C. Cells were incubated at 4 °C with an unconjugated antibody for SARS-CoV-2 nucleoprotein and an antibody to detect MAP-2^+^ neurons (Table 2) followed by a AF488-conjugated donkey anti-rabbit and AF647-conjugated donkey anti-guinea pig secondary antibody (Invitrogen) for 30 min. After washing steps, cells measured on a flow cytometer (BD FACSLyric).

### Multiplexed bead assay for cytokine profiling

Cytokines in the supernatant of the hiPSC-derived neural co-cultures were measured using the LEGENDplex human antivirus response panel (BioLegend). The kit was used according to the manufacturer’s manual with a small adaptation. After adding the SA-PE solution and performing the washing steps, the beads were fixed with 10% formalin for 30 min at room temperature and washed twice with the provided wash buffer. Afterwards cytokines were measure on a flow cytometer (BD FACSLyric).

### Reverse transcriptase quantitative PCR

For the measurement of gene expression by RT-qPCR, total RNA was isolated as described previously in Lamers et al. [46]. Briefly, 60 μL of sample was lysed in 90 μL of MagnaPure LC Lysis buffer (Roche) followed by a 30-min incubation with 50 μL Agencourt AMPure XP beads (Beckman Coulter). Beads were washed thrice with 70% ethanol on a DynaMag-96 magnet (Invitrogen) and eluted in 50 μL diethylpyrocarbonate treated water. In total 500 ng of RNA were reverse transcribed with SuperScript™ IV Reverse Transcriptase using Random Hexamer Primers according to the manufacturers protocol (Promega). Subsequently, gene expression was determined with SYBR GREEN PCR Mastermix (Applied Biosystems) according to the manufacturers protocol on a 7500 Real Time PCR Cycler (Applied Biosystems) with gene specific primers listed in Additional File 1: Table S2. Relative expression values were calculated with the 2^−ΔΔCT^ method and normalized to the average CT values of the housekeeping genes *Gapdh* and *Rpl18*. For quantification of SARS-CoV-2 specific RNA, a RT-qPCR targeting the E gene of SARS-CoV-2 was used as previously reported in Corman et al. [47] and Ct values were compared to a standard curve derived from a titrated D614G virus stock.

### Statistical analysis

Statistical differences between experimental groups were determined as described in the figure legends. P values of ≤ 0.05 were considered significant. Graphs and statistical tests were made with GraphPad Prism version 9. Figures were prepared with Adobe Illustrator CC2019, Adobe Photoshop CC2019 and Biorender.

## Results

### Omicron BA.1 has impaired neurotropism and neurovirulence compared to Delta and D614G in hiPSC-derived cortical neurons

**Fig. 1:**
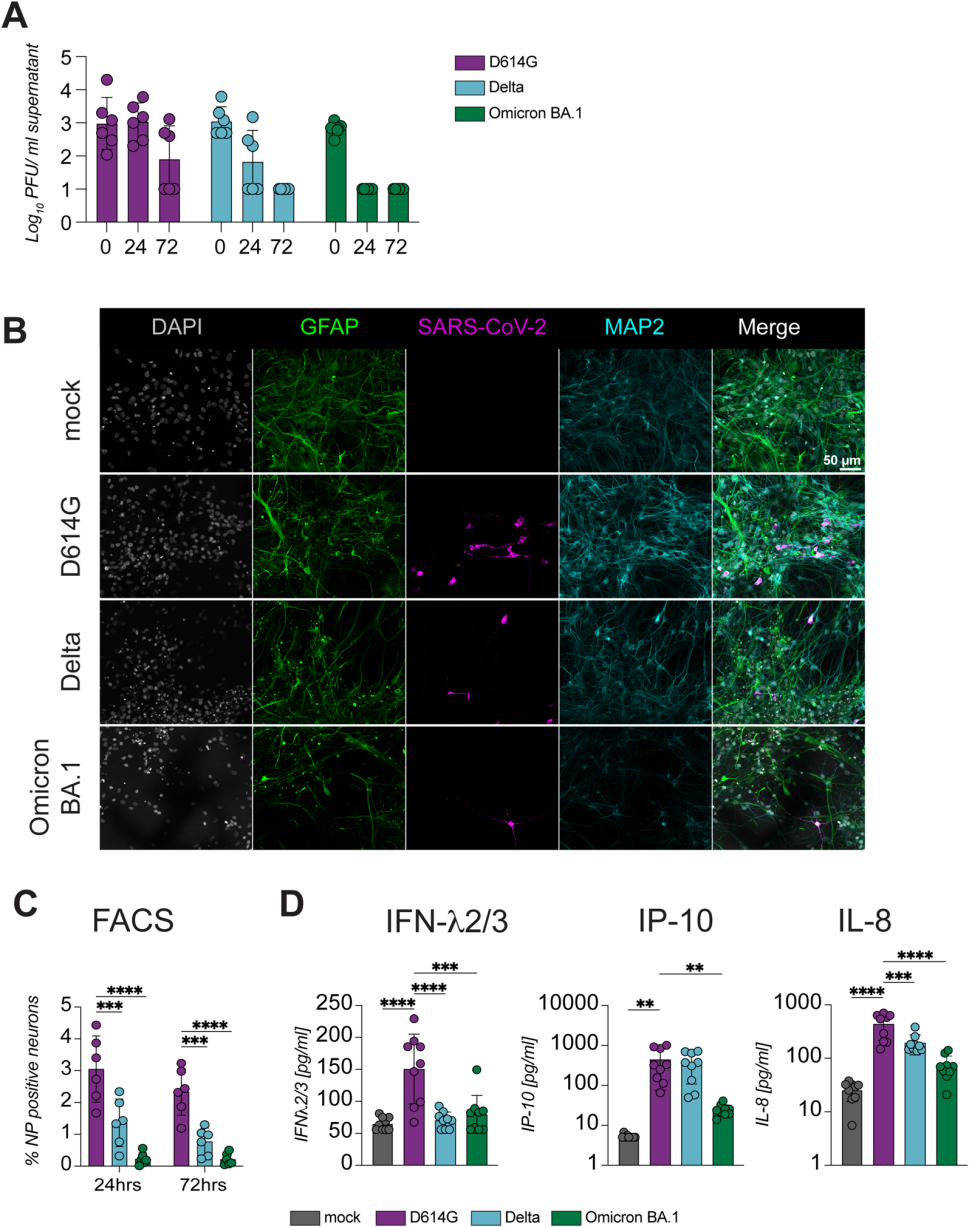
Omicron BA.1 has impaired neurovirulence and neurotropism compared to Delta and D614G. Human induced pluripotent stem cell-derived neural co-cultures consisting of cortical neurons and astrocytes were inoculated with the D164G, Delta and Omicron BA.1 variant at MOI 0.5. **A** Growth kinetics of D164G, Delta and Omicron BA.1. Growth curves were performed three independent times, once in biological duplicates and two times in biological triplicates. **B** Neural co-cultures were fixed at 24 h post infection. To investigate the cellular tropism co-cultures were stained with microtubule-associated protein (MAP2, cyan) as a marker for neurons, astrocytes were identified by staining for glial fibrillary acidic protein (GFAP) (green), and SARS-CoV-2 nucleoprotein (magenta) was used to identify infected cells. Cells were counterstained with Hoechst (grey) to visualize the nuclei. Data shown are representative examples from three independent experiments. **C** Percentage of SARS-CoV-2 infected MAP2^+^ neurons was calculated with flow cytometry at 24 and 72 h post infection. Data represent mean ± standard deviation (SD) from two independent experiments performed in biological triplicates. **D** Protein concentrations of IFN-λ2/3, IP-10 and IL-8 were measured in the supernatants of SARS-CoV-2 infected neural co-cultures with a multiplexed bead assay. The data are derived from three independent experiments, and each experiment was performed in biological triplicates. Statistical significance in **C** and **D** was calculated with a one-way analysis of variance (ANOVA) with a Bonferroni post hoc test, and the means from the mock-infected samples were compared to the means from the SARS-CoV-2 infected samples at 24 and 72 h post infection. Asterisks indicate statistical significance: *P < 0.05, **, *P* < 0.01, ***P < 0.001, ****P < 0.0001)

To evaluate the replication efficiency of the ancestral D614G, Delta and Omicron BA.1 variant, we used previously described hiPSC-derived co-cultures consisting of Ngn2 neurons co-cultured with hiPSC-derived astrocytes [35, 40, 41]. Ngn2 co-cultures were infected with the different SARS-CoV-2 variants at MOI 0.5. No efficient replication was observed in the neural co-cultures with any of the viruses (Fig. 1A). However, immunofluorescent staining for SARS-CoV-2 nucleoprotein at 24 and 72 h post inoculation (hpi) did reveal that D614G infected a higher proportion of cells than Delta and Omicron BA.1 (Figs. 1B and Additional File 1: Fig. S1). All infected cells were MAP2^+^ neurons. No infected GFAP^+^ astrocytes were observed. The percentage of infected MAP2^+^ neurons was significantly higher in D614G infected cultures (3%) compared to the Delta (1.4%) and Omicron BA. 1 (0.19%) variant (Fig. 1C). We previously determined that the SARS-CoV-2 D614G variant induced IFNλ2/3 and IL-8 [35]. Therefore, we measured cytokines in the supernatant of the neural co-cultures using a multiplexed bead assay to determine if differences in infection percentage also resulted in changes of the inflammatory response. D614G inoculation induced significantly higher levels of IP-10, IFNλ2/3 and IL-8 than inoculation with Delta or Omicron BA.1 (Fig. 1D). Taken together, these data suggest that Delta and Omicron BA.1 have reduced neurotropic and neurovirulent potential in neural co-cultures compared to the ancestral D614G strain.

### Neuroinvasiveness of D614G, Delta and Omicron BA.1.

**Fig. 2:**
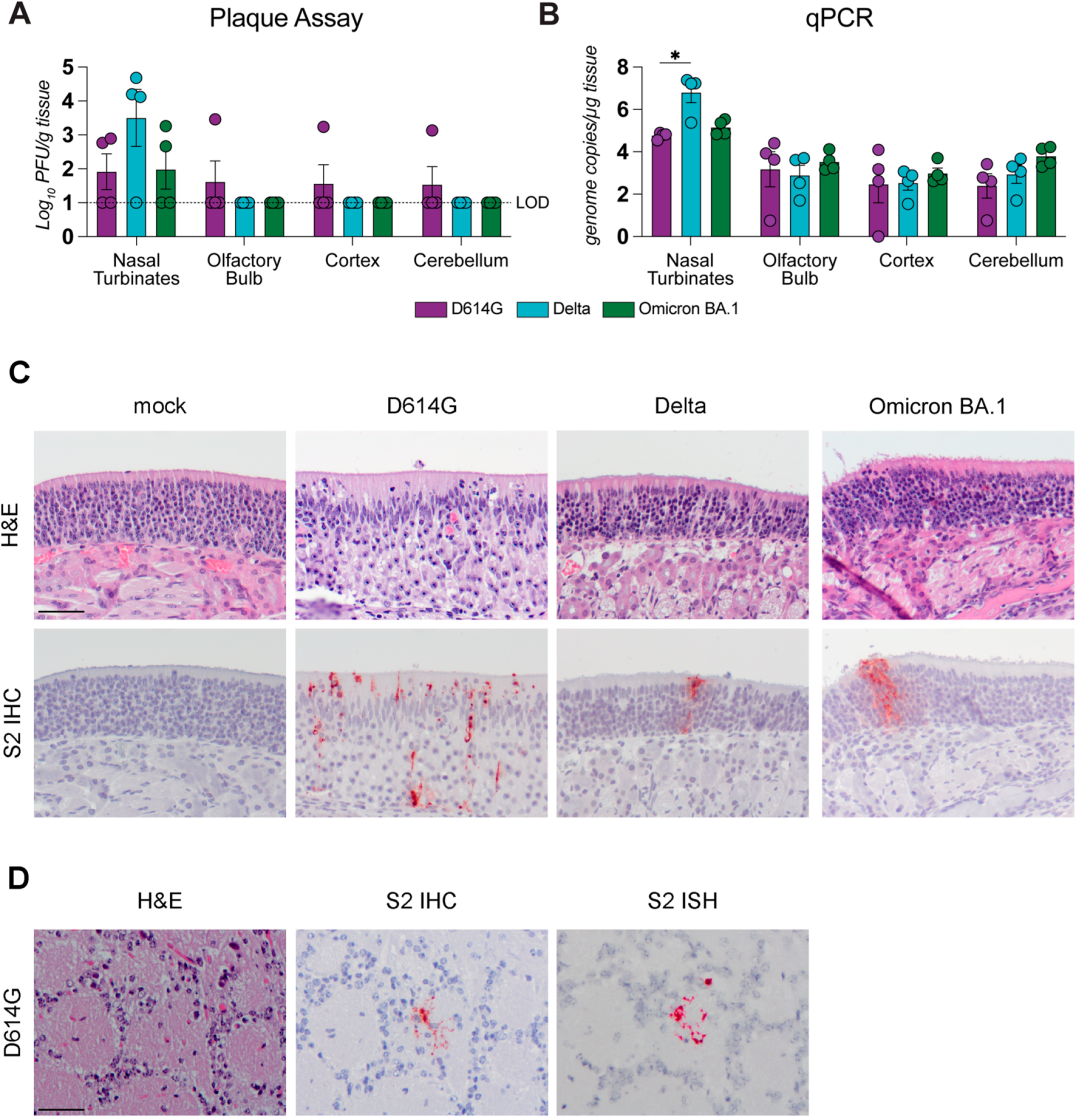
SARS-CoV-2 in nasal turbinates, olfactory bulb, cerebral cortex and cerebellum. Syrian golden hamsters were intranasally inoculated with 10^5^TCID_50_ D614G, 5.0 × 10^4^PFU Delta and Omicron BA.1 variant. Five days after inoculation, hamsters were sacrificed. Infectious virus titers (**A**) and viral copy RNA (**B**) in homogenates of nasal turbinates, olfactory bulb, cerebral cortex and cerebellum were quantified using plaque assay or reverse transcriptase quantitate PCR (RT-qPCR) respectively. Statistical significance was calculated with a Two-Way analysis of variance (ANOVA) with a Dunnett’s *posthoc* test. Averaged values of four individual animals per infection group were compared with all every other averaged value of four infected animals. Asterisks indicate statistical significance*, *P* < 0.05. LOD, limit of detection .(**C**) Hematoxylin and eosin (H&E) staining and SARS-CoV-2 nucleoprotein detected using immunohistochemistry (S2 IHC) in the olfactory mucosa of hamsters five days post exposure. **D** H&E staining SARS-CoV-2 nucleoprotein (S2 IHC) and SARS-CoV-2 viral RNA (S2 in-situ hybridization ISH) in the glomerular layer of the olfactory bulb in D614G inoculated hamsters

Nasal turbinates (containing olfactory mucosa), olfactory bulb, cerebral cortex and cerebellum were collected from hamsters five days post intranasal inoculation with SARS-CoV-2 variant D614G, Delta or Omicron BA.1 (Additional File 1: Fig. S2). No significant difference between the viral titers in the nasal turbinates was observed, although there was a non-significant trend of higher titers in hamsters infected with the Delta variant, compared to D614G and Omicron BA.1 (Fig. 2A). There was significantly more viral RNA in the nasal turbinates of hamsters inoculated with Delta compared to D614G (Fig. 2B). In the olfactory epithelium, SARS-CoV-2 antigen was most abundantly found with D614G inoculation compared to Delta and Omicron BA.1 (Fig. 2C and Additional File 1: Table S1). In the nasal turbinates, D614G inoculated hamsters showed the most prominent histopathological evidence of inflammation, as demonstrated by multifocal mild to moderate attenuation of olfactory epithelium colocalized with positive cytoplasmic immunohistochemical staining for SARS-CoV-2 antigen (Additional File 1: Table S1). Within the affected olfactory epithelium and lamina propria, predominantly neutrophilic infiltrates were present and the associated turbinates’ lumina contained substantial mucopurulent exudates. As previously reported by Rissmann et al. and others, [9, 48, 49] Delta or Omicron BA.1 inoculated hamsters exhibited fewer histological lesions compared to D614G in the respiratory tract. Omicron BA.1 inoculated hamsters showed the mildest inflammatory lesions within the olfactory mucosa.

Next, we determined the presence of virus, viral RNA and virus antigens in the olfactory bulb, cerebral cortex and cerebellum. In the olfactory bulb, infectious virus could be detected in about one-fourth of D614G inoculated animals while none was observed with Delta or Omicron BA.1 variant (Fig. 2A). However, similar levels of viral RNA were detected in the olfactory bulb in all groups (Fig. 2B). Virus antigen, detected by IHC, was found exclusively in the olfactory bulb in three of the four D614G infected animals (Additional File 1: Table S1). Based on tissue architecture, location and cellular characteristics, viral antigen was present in periglomerular cells of the glomerular layer, varying across animals from sparse individual periglomerular cells to several small clusters of cells represented in Fig. 2C. Viral RNA, detected by in-situ hybridization (ISH), was found at the same sites where SARS-CoV-2 antigen was detected using serial sections of the glomerular layers (Fig. 2D). No histological lesions were observed in the olfactory bulbs of any of the hamsters.

In the cerebral cortex, infectious virus was isolated from only one of the four D614G inoculated hamsters, without evidence for the presence of viral antigen or histological lesions in the cerebral cortex. In the cerebellum, infectious virus was isolated in only one of four D614G inoculated hamsters and none of the Delta or Omicron BA.1 infected hamsters, however there were no differences in viral RNA levels among the different groups. Similar to the cerebral cortex, there was no evidence for the presence of viral antigen or histological lesions in the cerebellum of any of the hamsters.

### Prominent antiviral and inflammatory response in the olfactory bulb of hamsters inoculated with D614G, but not Delta or Omicron BA.1

**Fig. 3:**
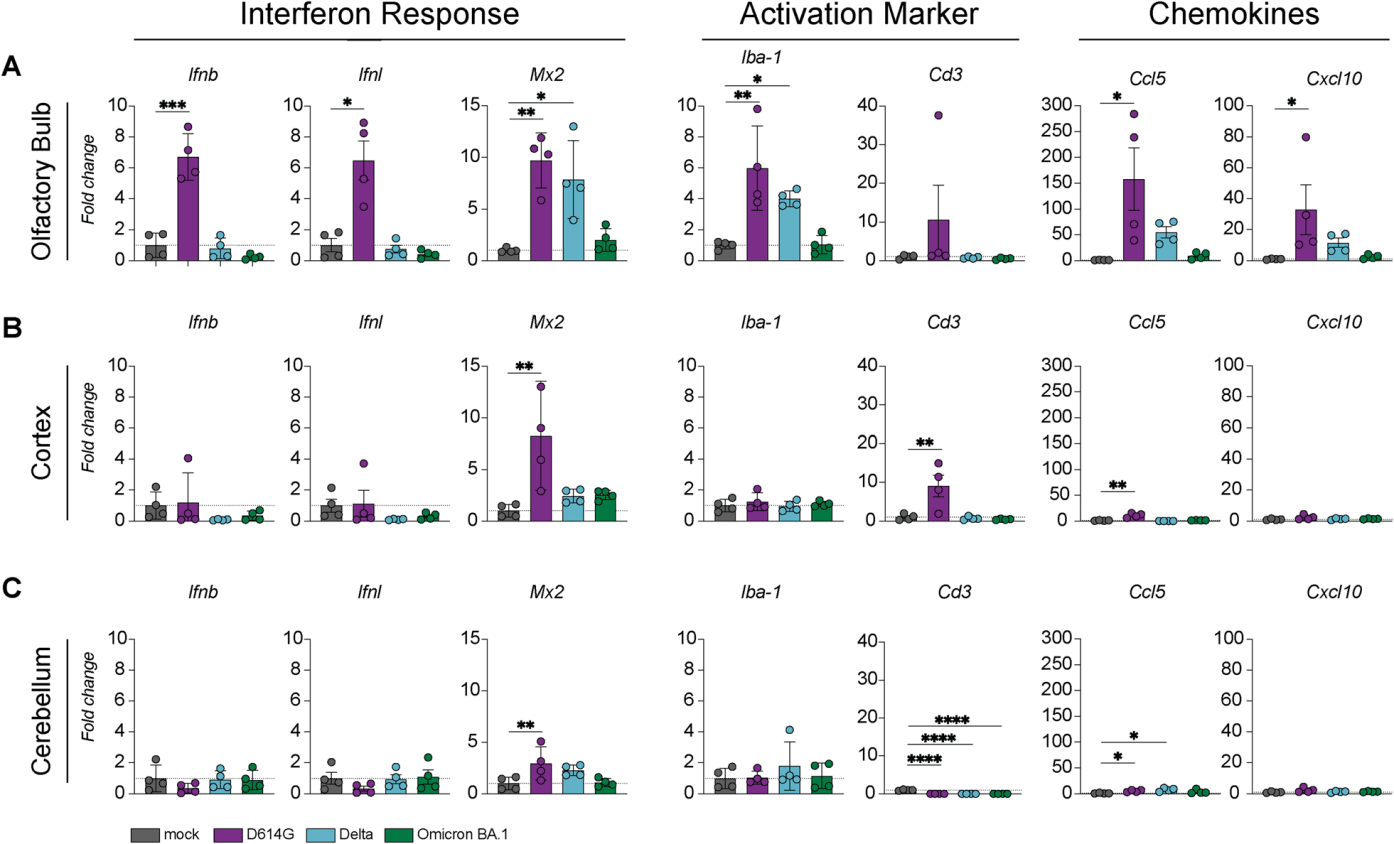
Antiviral and inflammatory responses in the olfactory bulb, cerebral cortex and cerebellum of SARS-CoV-2 infected hamsters. Expression of several genes important for interferon responses, for recruitment of microglia/macrophages and T-cells and chemokines were determined with reverse transcriptase quantitative PCR in the **A** olfactory bulb, **B** cerebral cortex and **C** cerebellum. The data displayed represent four animals per group. Statistical significance was calculated with a One-Way analysis of variance (ANOVA) with a Dunnett’s *posthoc* test. Averaged values of four individual animals per infection group were compared to values of four mock treated animals. Asterisks indicate statistical significance*, *P* < 0.05, **, *P* < 0.01, ***P < 0.001, ****P > 0.0001). Ifnb, interferon-β; Ifnl, interferon-λ; Mx2, interferon-induced GTP-binding protein; Aif1/Iba-1 allograft inflammatory factor 1 *Aif1* encoding the protein ionized calcium-binding adapter molecule 1 (IBA-1); Cd3, cluster of differentiation*; Cxcl10,* C-X-C motif chemokine 10; CCL5 C–C motif chemokine ligand 5 (*Ccl5)*

To quantify the antiviral response, we first examined the expression of type-I and type-III-interferon (IFN) in the olfactory bulb, cerebral cortex and cerebellum by reverse transcriptase quantitative PCR (RT-qPCR). Within the olfactory bulb, interferon-β (*Ifnb)* and interferon-λ (*Ifnl)* mRNA were upregulated in hamsters inoculated with D614G, but not Delta or Omicron BA.1 variant (Fig. 3A). Similarly, several canonical interferon-stimulated genes (ISGs) such as interferon-induced GTP-binding protein *Mx2,* interferon stimulated gene 15 (*Isg15)*, signal transducer and activator of transcription 1 (*Stat1)*, and interferon regulatory protein 7 (*Irf)* were upregulated in the olfactory bulbs of D614G, but not Delta or Omicron BA.1 infected hamsters (Fig. 3A and Additional File 1: Fig. S3A). Induction of IFNs and ISGs was observed exclusively in the olfactory bulb in contrast to the cerebral cortex or cerebellum (Fig. 3B, C, Additional File 1: Figs. S3B and S3C).

Next, we investigated the inflammatory response in the olfactory bulb, cerebral cortex and cerebellum. A significant increase of allograft inflammatory factor 1 *Aif1* mRNA, encoding the protein ionized calcium-binding adapter molecule 1 (IBA-1), was detected in the olfactory bulbs of hamsters inoculated with either D614G or Delta, but not in the hamsters inoculated with the Omicron BA.1 variant (Fig. 3B). The T cell associated gene cluster of differentiation 3 (*Cd3)* was not upregulated in the olfactory bulb of any of the groups. In the olfactory bulb of D614G infected hamsters, but not in hamsters inoculated with Delta or Omicron BA.1, a significant increase of the chemokines C-X-C motif chemokine 10 (*Cxcl10)* and C-C motif chemokine ligand 5 (*Ccl5)* mRNA was detected*.* In the cerebral cortex, *Cd3* expression was upregulated exclusively in D614G inoculated hamsters. In the cerebellum, there was no evidence for the induction of ISGs, proinflammatory cytokines or recruitment markers for macrophages/microglia (Fig. 3B and Additional File 1: Fig. S3B).

Together, these data suggest that antiviral and inflammatory responses are predominantly located in the olfactory bulb five days post SARS-CoV-2 inoculation. This response was most prominent in hamsters inoculated with D614G. In the Delta inoculated hamsters, we detected an upregulation of the ISG *Mx2* and the inflammatory marker *Iba-1* in the olfactory bulb. No antiviral or inflammatory response was detected in the olfactory bulb of hamsters inoculated with the Omicron BA.1 variant.

### Activation of microglia/macrophages in the olfactory bulb of D614G- but not in Delta- or Omicron BA.1 inoculated hamsters

**Fig. 4:**
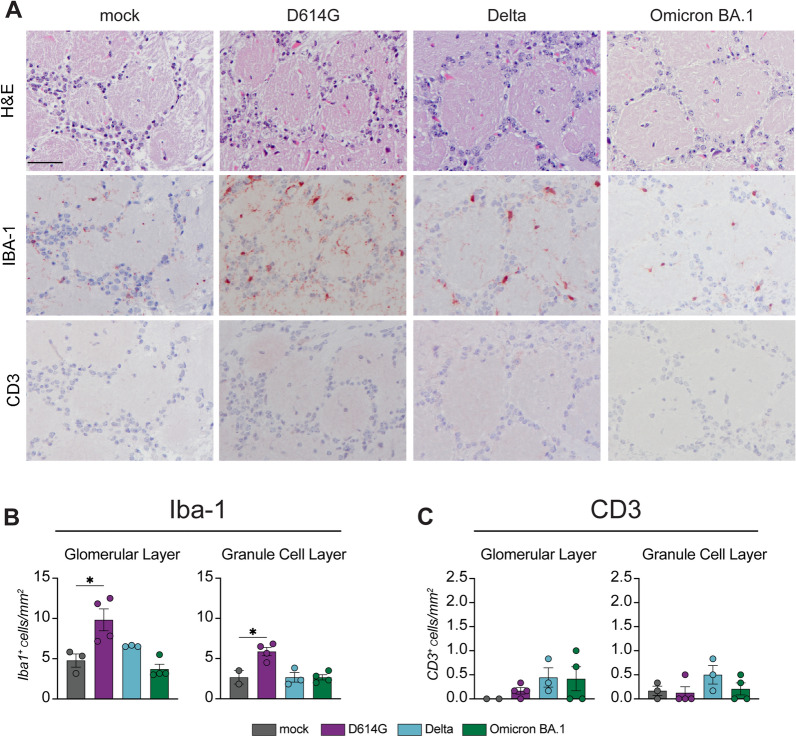
D614G infection but not Delta or Omicron BA.1 infection increases the number of Iba-1^+^ microglia/macrophages in the olfactory bulb. **A** Hematoxylin and eosin (H&E) staining of the glomerular layer in the olfactory bulb. Detection of Iba1^+^ cells and CD3^+^ cells in the glomerular layer of the olfactory bulb. Counting of **B** Iba-1^+^ cells and **C** CD3.^+^ cells in the glomerular layer and the granular cell layer of the olfactory bulb. Statistical significance was calculated with a One-Way analysis of variance (ANOVA) with a Dunnett’s *posthoc* test. Averaged values of four individual animals per infection group were compared to values of four mock treated animals. Asterisks indicate statistical significance*, *P* < 0.05)

In order to confirm the increase of *Iba-1* mRNA expression in the olfactory bulb of hamsters inoculated with D614G, we analysed IBA-1 antigen expression by IHC in the different layers of the olfactory bulbs of the D614G, Delta or Omicron BA.1 inoculated hamsters (Additional File 1: Fig. S4). We detected IBA-1 expression throughout the different layers of the olfactory bulb in all animals examined, including those with mock inoculation (Fig. 4A and Additional File 1: Fig. S3). In all D614G inoculated hamsters, the number and morphology of IBA-1 expressing microglia increased. An increase of IBA-1^+^ cells was observed in both the glomerular and granule cell layers of D614G infected hamsters, but not Delta or Omicron BA.1 inoculated hamsters (Fig. 4B). IBA-1^+^ cells were more frequently clustered around small blood vessels throughout all layers in D614G inoculated hamsters without any evidence for histological changes based on the HE stainings. (Additional File 1: Fig. S6). At this point, we cannot differentiate if the observed IBA-1^+^ cells around the blood vessels are activated microglia or infiltrating macrophages. In accordance with *Cd3* mRNA expression, we did not observe and increase of CD3^+^ cells in any of the hamsters (Fig. 4C).

## Discussion

This study indicates notable differences in the neuroinvasiveness, neurotropism and neurovirulence between the ancestral D614G and the Delta and Omicron BA.1 SARS-CoV-2 variants using in vitro hiPSC derived neural cultures and in vivo Syrian golden hamster model. The hamster model has been proven useful for studying the pathogenesis of respiratory disease caused by infection with different SARS-CoV-2 variants [9, 25–28, 50]. Methodologically, our current findings also confirm the value of parallel investigations of the neuroinvasiveness and neurovirulence of different SARS-CoV-2 variants in animal models and hiPSC-derived models.

Our findings are in accordance with previous reports that the ancestral SARS-CoV-2 D614G variant can be neuroinvasive, entering the CNS via the olfactory nerve [26–28, 51]. In contrast, although viral RNA was detected in the olfactory bulbs by RT-qPCR in Delta and Omicron BA.1 inoculated hamsters, neither viral proteins nor RNA could be detected by IHC or ISH, respectively. This suggests a reduced neuroinvasive potential of these variants compared to the ancestral D614G variant, although we cannot exclude the possibility of neuroinvasion by the Delta and Omicron BA.1 variant at earlier timepoints following inoculation. However, previous finding in hACE-2 transgenic mice have also shown that Omicron BA.1 is less neuroinvasive than earlier variants [21]. As viral antigen and virus induced lesions were more abundant in the olfactory mucosa of D614G inoculated hamsters compared to Delta or Omicron BA.1 inoculated hamsters, an association might exist between replication efficiency in the olfactory mucosa and detection of virus antigen and viral RNA in the olfactory bulb. Notably, such a pattern is not only observed with SARS-CoV-2 [10–12, 21] but has previously been reported for influenza A viruses, in which highly pathogenic H5N1 virus replicates efficiently in the olfactory mucosa and spreads to the CNS via the olfactory nerve [52–54], whereas, seasonal and pandemic influenza viruses replicate less efficiently in the olfactory mucosa, and do not spread—at least not efficiently—to the CNS via the olfactory nerve [53, 55].

D614G appeared to be more neurotropic and neurovirulent than the Delta and Omicron BA.1 variant both in our in vitro and in vivo models. The observed neurovirulence associated with D614G infection included antiviral and inflammatory responses, which in the hamster model were primarily observed in the olfactory bulb and to a lesser extent cerebral cortex and cerebellum, which fits with previous findings [27, 56]. On the contrary, infection with Omicron BA.1 variant induced only very minimal neuroinflammation*,* where the Delta variant showed an intermediate neuroinflammation phenotype in vitro and in vivo. Accordingly, it is likely that the neuroinflammation observed with D614G is associated with its ability to replicate efficiently in the olfactory mucosa and/or its ability to invade the CNS.

The decreased pathogenicity of Omicron BA.1 might be associated with newly acquired molecular alterations such as a heavily mutated receptor binding domain of the Spike protein which modulates cell entry[57, 58], as well as mutations in non-structural proteins potentially altering replication kinetics [59]. Genotypic changes in the receptor binding site of the Spike protein often correspond with phenotypic differences in ACE-2 receptor affinities, and some studies have already shown that there are differences in the entry process and fusogenicity among SARS-CoV-2 variants [57, 58]. It is likely that these differences in the Spike proteins between the various SARS-CoV-2 variants influence for example their tropism within the olfactory mucosa which might impact the neuroinvasiveness.

How our observations contribute to a deeper understanding of the pathophysiological mechanism underlying human clinical neurological complications remains to be established. However, the observed abundant replication of D614 within the olfactory mucosa and the associated lesions likely contribute to anosmia. Interestingly, anosmia was observed more frequently in humans after infection with viruses containing the D614G mutation early in the pandemic [60] and recent findings suggest that anosmia occurs less frequently after infection with Omicron BA.1 compared to earlier variants [9–12]. Although these findings require replication and independent validation, these observations are in accordance with the less efficient replication of Omicron BA.1 in the olfactory mucosa compared to D614G [18, 61]. Whether the observed neuroinflammation in the D614G inoculated hamsters contributes to anosmia or other neurological manifestations [27], or whether this is a protective response preventing subsequent virus spread throughout the CNS, also warrants further investigation across the acute and post-acute stages of illness.

## Supplementary Information

**Additional file 1.** Figure S1-S6 and Table S1-S2.
